# Sequential activation of conventional and plasmacytoid dendritic cells in autoimmune pancreatitis and systemic lupus erythematosus: similarities and dissimilarities

**DOI:** 10.3389/fimmu.2025.1554492

**Published:** 2025-02-18

**Authors:** Akane Hara, Tomohiro Watanabe, Kosuke Minaga, Ken Kamata, Warren Strober, Masatoshi Kudo

**Affiliations:** ^1^ Department of Gastroenterology and Hepatology, Kindai University Faculty of Medicine, Osaka-Sayama, Japan; ^2^ Mucosal Immunity Section, Laboratory of Clinical Immunology and Microbiology, National Institute of Allergy and Infectious Diseases, National Institutes of Health, Bethesda, MD, United States

**Keywords:** autoimmune pancreatitis, conventional dendritic cells, plasmacytoid dendritic cells, systemic lupus erythematosus, type I IFNs

## Abstract

Type 1 autoimmune pancreatitis (AIP) and systemic lupus erythematosus (SLE) are caused by type I IFNs secreted by plasmacytoid dendritic cells (pDCs). Our understanding of the immune consequences before and after pDC activation in SLE is expanding, whereas knowledge on those in AIP are insufficient. In this article, we summarize the similarities and dissimilarities in pDC activation between AIP and SLE. In SLE, neutrophil extracellular traps containing self-DNA, anti-microbial peptides, and endogenous alarmins form anti-DNA antibody complexes, promoting type I IFN production by pDCs. Type I IFNs produced by pDCs function as initiators rather than effectors in SLE, as evidenced by the fact that these cytokines induce the maturation of conventional DCs (cDCs) leading to the expansion of autoreactive T cells and B cells. Notably, type I IFNs produced by pDCs were observed at the maturation phase but not at the induction phase in experimental AIP. Mechanistically, cDCs producing type I IFNs, C-X-C motif chemokine ligand 9 (CXCL9), and CXCL10 are initiator cells of AIP, and C-X-C chemokine receptor 3 (CXCR3)^+^T helper type 1(Th1) cells migrate to the pancreas in response to CXCL9 and CXCL10. CXCR3^+^Th1 cells produce C-C chemokine ligand 25 (CCL25) to attract C-C chemokine receptor 9 (CCR9)^+^pDCs to the pancreas. Pancreatic pDCs producing type I IFNs, CXCL9, CXCL10, and CXCR3^+^Th1 cells producing CCL25 form a positive feedback loop in which the sensing of intestinal dysbiosis induces large amounts of type I IFNs by pDCs.

## 1 Introduction

Dendritic cells (DCs) are specialized immune cell subsets that bridge the innate and adaptive immune systems (1). DCs recognize pathogens and alarmins by activating pattern recognition receptors (PRRs), including Toll-like receptors (TLRs), and present microbial and endogenous antigens (Ags) to T cells (1, 2). Thus, DCs produce proinflammatory cytokines upon recognition of microbe-associated molecular patterns (MAMPs) and damage-associated molecular patterns (DAMPs) through PRRs. Proinflammatory cytokines including type I IFNs, IL-1β, IL-6, IL-12, IL-23, and TNF-α produced by DCs play indispensable roles in the differentiation of T helper type 1 (Th1), Th2, and Th17 cells (1, 2). Besides proinflammatory cytokines, DCs activate CD4^+^ T cells via cognate interactions with class II and costimulatory molecules (1, 2). These unique properties enable DCs to play a central role in host defense against invading pathogens (1, 2). In contrast, excessive secretion of proinflammatory cytokines and presentation of endogenous Ags by DCs cause autoimmunity (1).

DCs can be classified into two major types: conventional DCs (cDCs) and plasmacytoid DCs (pDCs). cDCs expressing high class II levels are potent in Ag presentation and secretion of proinflammatory cytokines triggered by TLR2, TLR3, TLR4, TLR5, TLR7, and TLR9 (3, 4). pDCs are a specialized DC type with the ability to produce type I IFNs (IFN-α and IFN-β) upon sensing microbial nucleic acids through TLR7 and/or TLR9 (5, 6). Type I IFN responses by activation of pDCs are indispensable components of viral host defenses; however, recognition of self-or microbial nucleic acids by pDCs sometimes causes autoimmunity in a type I IFN-dependent manner (5, 6). Excessive production of type I IFNs has been implicated in the pathogenesis of systemic lupus erythematosus (SLE) and type 1 autoimmune pancreatitis (AIP) (5, 6). The pathogenicity of pDC-derived type I IFNs in autoimmunity has been verified in recent clinical trials, wherein neutralization of type I IFN receptor subunit 1 by anifrolumab or pDC-specific blood dendritic cell antigen 2 (BDCA2) by litifilimab led to clinical remission in patients with active SLE (7, 8). In addition, serum concentrations of type I IFNs are higher in patients with SLE and AIP than in patients with other autoimmune diseases or in healthy controls (5, 9, 10). Therefore, the activation of pDCs that produce type I IFNs is undoubtedly involved in the immunopathogenesis of SLE and type 1 AIP. However, cDCs are professional Ag-presenting cells that can present endogenous and exogenous Ags and produce large amounts of proinflammatory cytokines upon TLR activation. Previous studies have placed too much emphasis on the function and pathogenicity of pDCs in autoimmunity. Recent studies have shown that cDCs function as initiators or effectors in AIP and SLE, both upstream and downstream of pDCs.

In this mini-view, we focus on the interaction between cDCs and pDCs in the development of type 1 AIP and SLE. We identified a positive cytokine/chemokine feedback loop produced by cDCs, C-X-C chemokine receptor 3 (CXCR3)^+^Th1 cells, and pDCs, in the development of type 1 AIP (10). In SLE, cDCs, which mature in response to pDC-derived type I IFNs, induce the expansion of autoreactive T and B cells through Ag presentation to these cells (11).

## 2 Activation of pDCs in type 1 AIP and SLE

AIP is classified into type 1 and type 2 AIP, with the former type of AIP being a pancreatic manifestation of systemic IgG4-related disease (IgG4-RD) (12). Over 90% of AIP cases are considered type 1 AIP; type 2 AIP is rare. IgG4-RD is a systemic chronic fibroinflammatory disorder characterized by elevated serum IgG4 levels and the infiltration of IgG4-expressing plasmacytes into the affected organs (6, 12). Another prominent feature of type 1 AIP and IgG4-RD is the presence of synchronous and metachronous lesions in multiple organs, including the pancreas, salivary glands, kidneys, and lungs (6, 12). Despite newly discovered autoimmune disorders, our understanding of pathogenic adaptive immunity associated with these disorders is expanding. Laminin 511 and annexin A11 have been identified as autoAgs in type 1 AIP and IgG4-RD (13, 14). In addition, clonally expanded cytotoxic CD4^+^ T cells, that produce proinflammatory and profibrogenic cytokines (IFN-γ, IL-1β, and TGF-β) as well as cytolytic factors (granzyme and perforin), mediate tissue injury in IgG4-RD (15). Compared with that of adaptive immunity, our knowledge of innate immunity causing chronic fibroinflammatory responses in type 1 AIP is limited.

**Figure 1:**
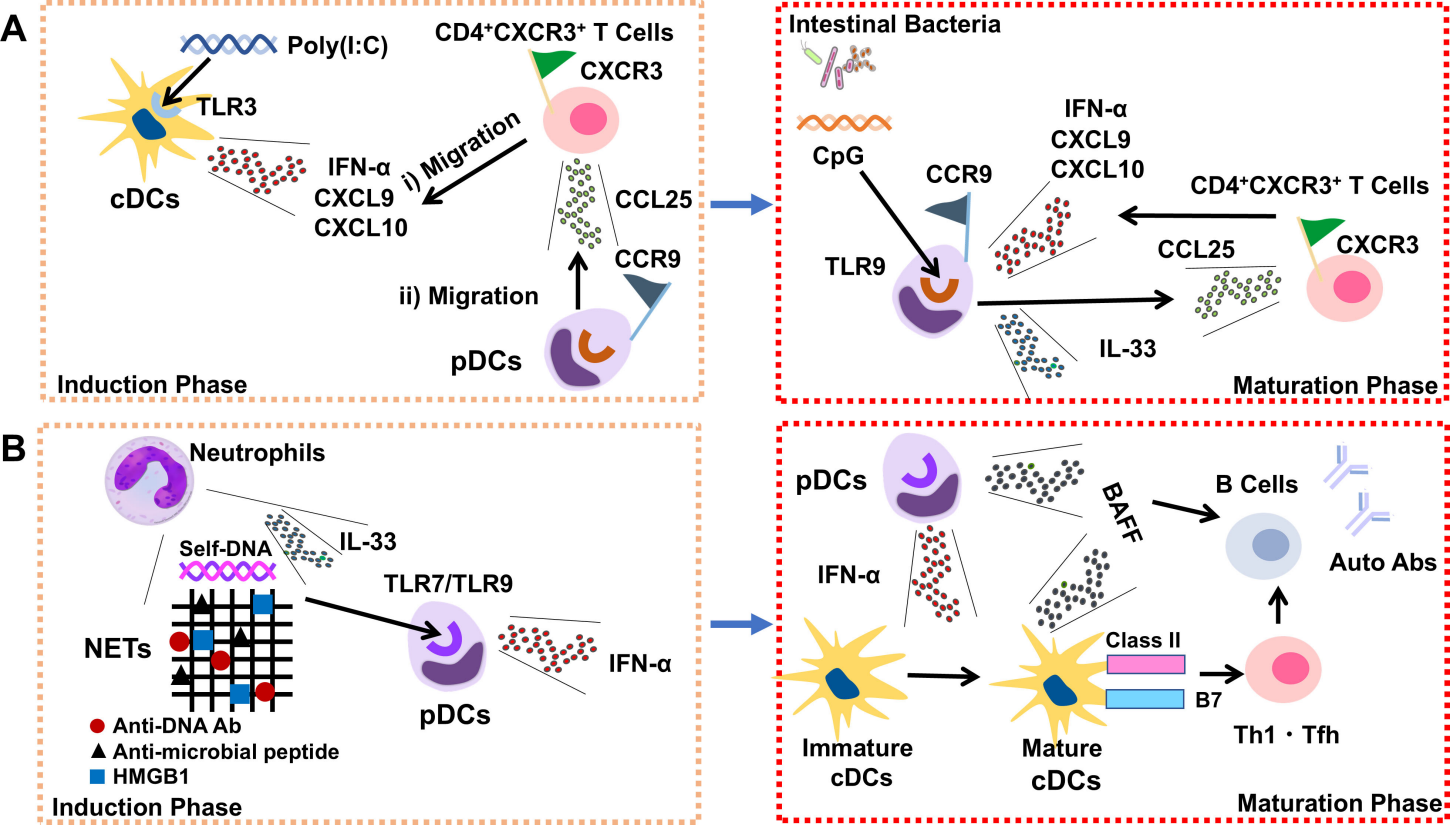
Consequences of activation of conventional and plasmacytoid dendritic cells in autoimmune pancreatitis and systemic lupus erythematosus. **(A)** Activation of conventional and plasmacytoid dendritic cells in autoimmune pancreatitis. Conventional dendritic cells (cDCs) residing in the non-inflamed pancreas produce type I IFNs, C-X-C motif chemokine ligand 9 (CXCL9), and CXCL10 upon sensing polyinosinic-polycytidylic acid [poly(I:C)] by toll-like receptor 3 (TLR3). C-X-C chemokine receptor 3 (CXCR3)^+^T helper type 1 (Th1) cells, that migrate to the pancreas in response to CXCL9 and CXCL10, produce C-C motif chemokine ligand 25 (CCL25) (left panel). Plasmacytoid dendritic cells (pDCs) expressing the C-C chemokine receptor 9 (CCR9) migrate to the pancreas in response to CCL25 and produce large amounts of type I IFNs upon sensing intestinal dysbiosis by TLR9. A positive cytokine/chemokine feedback loop in which the interaction between CXCR3^+^Th1 cells and pDCs increases the production of CCL25, CXCL9, CXCL10, and type I IFNs causes further rounds of pancreatic pathogenic T cell and pDC accumulation (right panel). **(B)** Consequences of cDCs and pDCs activation in patients with SLE. Neutrophil extracellular traps (NETs) derived from activated neutrophils include self-DNA, the anti-microbial peptide LL37, and high mobility group box 1 (HMGB1). NETs form a complex with an anti-DNA antibody and IL-33 to activate TLR7 and TLR9 in pDCs. TLR7 and TLR9 activation in pDCs induces the production of type I IFNs (left panel). Type I IFNs produced by pDCs induce adaptive and innate immune responses. These include the maturation of cDCs expressing high levels of costimulatory and class II molecules. Antigen presentation by mature cDCs facilitates the expansion of autoreactive T and B cells. Type I IFNs also induce the differentiation of Th1 and T follicular helper (Tfh) cells as well as B cell-activating factor (BAFF) production by pDCs and cDCs (right panel).

Repeated injection of polyinosinic-polycytidylic acid (poly(I:C), a TLR3 ligand) into MRL/MpJ mice led to AIP development, characterized by acinar architecture destruction and immune cell infiltration (16, 17). MRL/MpJ mice treated with poly(I:C) manifested autoimmune sialadenitis, cholangitis, and nephritis in addition to AIP; thus, this model shares pathological findings with human type 1 AIP and IgG4-RD (16, 17). Extensive flow-cytometric analyses utilizing pancreatic immune cells from MRL/MpJ mice displaying AIP revealed that the pancreatic accumulation of pDCs producing type I IFNs and IL-33 is one of the prominent features of this experimental AIP (16, 17) (**Figure 1A**). Blockade of type I IFN- or IL-33-mediated signaling pathways by systemic administration of anti-type I IFN αβ receptor antibody (Ab) or anti-IL-33 receptor Ab markedly inhibits the development of experimental AIP (16, 17). Clinical relevance regarding these findings comes from the fact that serum IFN-α and IL-33 concentrations are markedly higher in patients with type 1 AIP and IgG4-RD than in patients with chronic alcoholic pancreatitis and healthy controls and that pDCs producing IFN-α and IL-33 are localized in the pancreas of patients with these disorders (9, 16, 17).

Intestinal dysbiosis has been implicated in the activation of pDCs that cause type 1 AIP (18, 19). Next-generation sequencing (NGS) analyses for the visualization of fecal microbiota composition revealed a marked difference between MRL/MpJ mice treated with and without poly(I:C). Bowel sterilization by oral administration of various antibiotics markedly suppressed the development of AIP, which effects were associated with diminished accumulation of pDCs producing IFN-α and IL-33 (18–20). The degree of AIP was parallel to the injection dose of poly(I:C) in MRL/MpJ mice; 16 injections of 100 µg poly(I:C) caused severe AIP, whereas injections of 10 µg caused mild AIP (18, 19). Taking advantage of the poly(I:C) dose dependency, a fecal microbiota transplantation (FMT) study was conducted. As expected, MRL/MpJ mice treated with 10 µg poly(I:C) exhibited a mild degree of AIP upon FMT from healthy mice. Notably, MRL/MpJ mice treated with 10 µg poly(I:C) exhibited a severe degree of AIP upon FMT from mice displaying severe AIP through pancreatic accumulation of pDCs producing IFN-α and IL-33 (18). Given that FMT alone from severe AIP mice did not cause mild or severe pancreatitis if mice did not receive poly(I:C) injection, these studies have suggested that intestinal dysbiosis enhances sensitivity to AIP through activation of pDCs producing IFN-α and IL-33.

The above-mentioned studies exploring the association between intestinal dysbiosis and AIP did not identify any pathogenic bacteria. Drinking of dextran sodium sulfate (DSS) allows bacterial translocation due to disruption of intestinal barrier integrity (21). We hypothesized that disruption of the intestinal barrier by DSS acts in concert with poly(I:C) and worsens experimental AIP through increased migration of intestinal bacteria into the pancreas (20). Combined treatment with DSS drinking and systemic poly(I:C) injection leads to the development of more severe AIP than poly(I:C) treatment alone (20). NGS analyses of pancreatic and fecal samples revealed that *Staphylococcus sciuri* migrates from the gastrointestinal tract to the pancreas upon disruption of the intestinal barrier, and functions as a pathobiont (20). Furthermore, mono-colonization with *S. sciuri* increased the sensitivity to experimental AIP through activation of pDCs producing IFN-α and IL-33. Collectively, these studies provide evidence that sensing intestinal dysbiosis by pDCs mediates AIP via the production of type I IFNs and IL-33. In human type 1 AIP, induction of remission by prednisolone is accompanied by reduction in gut colonization of *Klebsiella pneumoniae* (19). Although association between *K. pneumoniae* colonization and severity of pathology has not been established in human AIP, colonization with *K. pneumoniae* strains is associated with the severity of inflammatory bowel diseases across geography (22). Therefore, this bacterium might act as a common pathobiont for both AIP and inflammatory bowel diseases.

The pivotal roles played by pDCs in type 1 AIP are reminiscent of SLE, in that neutralization of type I IFN signaling pathways or depletion of pDCs is effective (7, 8). Although type I IFN production by pDCs is one of the most critical components driving the development of both type 1 AIP and SLE, the activation mechanisms and functions of pDCs differ in several aspects. First, pDCs associated with type 1 AIP produce large amounts of type I IFNs upon recognition of TLR9 ligands (CpG) derived from bacteria, whereas NETosis-derived self-DNAs together with DAMPs (anti-microbial peptide LL37 and high mobility group box 1) accelerate type I IFN signaling through activation of TLR7 and/or TLR9 in SLE-associated pDCs (**Figure 1B**) (5, 6, 10, 18–20). Thus, the inducers of type I IFN production by pDCs are different between type 1 AIP and SLE: bacterial signals in type 1 AIP versus endogenous signals in SLE. Another difference between type 1 AIP and SLE is the cellular source of IL-33. pDCs produce IL-33 in a type I IFN-dependent manner in AIP to mediate tissue fibrosis (17). In SLE, IL-33 derived from neutrophils forms a complex with neutrophil extracellular traps (NETs) to facilitate type I IFN production by pDCs (23). Thus, IL-33 functions as a downstream and upstream cytokine of pDC-derived type I IFNs in patients with AIP and SLE, respectively. Collectively, type 1 AIP and SLE share pDC-derived type I IFNs as pathogenic cytokines. However, dissimilarities exist in the activators and functions of pDCs.

## 3 Interaction between pDCs and cDCs in type 1 AIP

We recently identified a cascade of immune mechanisms and consequences that lead to the activation of pDCs producing type I IFNs in AIP (**Figure 1A**) (10). MRL/MpJ mice developed full-blown AIP after completing 16 injections of poly(I:C). Mature AIP is characterized by pancreatic accumulation of pDCs producing type I IFNs, as mentioned above (16, 17). Kinetic studies addressing the timing of pDC accumulation have revealed that the percentage of pancreatic pDCs only mildly increased after three injections of poly(I:C) and reached a maximum level after 16 injections (10). Thus, Ag-presenting cells other than pDCs mediated experimental AIP during the induction phase.

Although poly(I:C) is a conventional TLR3 ligand, pancreatic pDCs, defined as PDCA-1^+^B220^low^ cells using flow-cytometry lack this TLR (10). In contrast, pancreatic cDCs, defined as CD11c^+^class II^high^ cells, express intracellular TLR3. Pancreatic cDCs, but not pDCs isolated from mice during the induction phase of experimental AIP, produce large amounts of type I IFNs, C-X-C motif chemokine ligand 9 (CXCL9), and CXCL10 upon stimulation with poly(I:C) (10). Thus, TLR3-expressing cDCs residing in the non-inflamed pancreas are AIP initiators that produce type I IFNs, CXCL9, and CXCL10. Detailed analyses of chemokines and chemokine receptor interactions have shown that CD4^+^CXCR3^+^Th1 cells migrate to the pancreas in response to CXCL9 and CXCL10, both of which are produced by cDCs during the initial phase (10). CD4^+^CXCR3^+^Th1 cells that migrate to the pancreas during the initial phase of AIP are unique in that they are potent producers of C-C chemokine ligand 25 (CCL25) and attract C-C chemokine receptor 9 (CCR9)-expressing cells. Adaptive CD4^+^CXCR3^+^Th1 cells attract CCR9^+^pDCs through the CCL25-CCR9 interaction. Importantly, the interaction between CD4^+^CXCR3^+^Th1 cells producing CCL25 and CCR9^+^pDCs producing type I IFNs is bidirectional because type I IFNs produced by pDCs enhance the secretion of CCL25 by T cells, which in turn facilitates CCR9^+^pDC accumulation into the pancreas. Furthermore, pDCs secretion of type I IFNs mediated by TLR9 activation was markedly augmented in the presence of CD4^+^CXCR3^+^Th1 cells, suggesting that activated CD4^+^CXCR3^+^Th1 cells act synergistically with bacterial DNA to differentiate pancreatic CCR9^+^pDCs that cause AIP.

In summary, we identified cytokine and chemokine networks comprising cDCs, CD4^+^CXCR3^+^Th1 cells, and pDCs (**Figure 1A**). Pancreatic cDCs that recognize poly(I:C) via TLR3 initiate inflammation by producing type I IFNs, CXCL9, and CXCL10. CD4^+^CXCR3^+^Th1 cells migrate to the pancreas in response to CXCL9 and CXCL10 produced by cDCs and function as hub cells bridging cDCs and pDCs. These T cells attract effector cells for AIP, that is pDCs, through the production of CCL25. CCR9^+^pDCs that migrate to the pancreas towards CCL25 secreted by CD4^+^CXCR3^+^Th1 cells play an indispensable role in developing mature AIP through the production of large amounts of type I IFNs. Such trains of events set up a positive feedback loop in which pDCs produce type I IFNs, CXCL9, and CXCL10 upon recognizing bacterial DNA via TLR9, thereby promoting further rounds of migration of CD4^+^CXCR3^+^Th1 cells and pDCs into the pancreas (10). Therefore, our study elucidated the details of the innate and adaptive immune responses upstream of effector pDC activation and identified sequential activation of cDCs, CD4^+^CXCR3^+^Th1 cells, and pDCs underlying the immunopathogenesis of pDC-driven AIP (10). This scenario may also apply to the immunopathogenesis of human type 1 AIP because serum IFN-α, CCL25, CXCL9, and CXCL10 concentrations are significantly higher in patients with type 1 AIP than in healthy controls and those with chronic alcoholic pancreatitis. In addition, serum IFN-α, CCL25, CXCL9, and CXCL10 concentrations are parallel to disease activity in patients with type 1 AIP (10).

## 4 Interaction between pDCs and cDCs in SLE

As mentioned previously, cDCs are initiator cells that trigger the formation of a positive cytokine and chemokine feedback loop comprising of CCL25 and type I IFNs derived from CD4^+^CXCR3^+^Th1 cells and pDCs, respectively, in experimental and human AIP (10). Activation of cDCs is also involved in the pathogenesis of SLE, as shown by the finding that both cDCs and pDCs contribute to the generation of a heightened IFN-stimulated gene signature in peripheral blood at the single-cell level (24). In contrast to cDC activation followed by the robust production of type I IFNs in AIP, the activation of pDCs producing type I IFNs precedes cDC activation in SLE (11) (**Figure 1B**). Strong evidence regarding the importance of pDC activation at the initial phase of the disease comes from studies employing BXSB/MpJ lupus-prone mice, in which pDCs can be selectively depleted by the administration of diphtheria toxin (DT). Rowland et al. generated BXSB-DT receptor (BXSB-DTR) mice by backcrossing BDCA2-DTR transgenic mice with BXSB/MpJ lupus-prone mice (25). Selective pDC depletion by DT administration at an early time point ameliorated lupus nephritis and reduced serum concentrations of autoAbs. The amelioration of autoimmunity by the early depletion of pDCs was accompanied by a marked reduction in T cells, B cells, and class II^+^ cDCs in the spleen, suggesting that type I IFN production by pDCs precedes cDC activation in SLE (25). The roles played by type I IFNs as SLE initiators have been confirmed by longitudinal studies of SLE, in which elevations in serum type I IFN concentrations precede diagnosis (26, 27).

Type I IFNs produced by pDCs exert various effects on both innate and adaptive immunity (28). Differentiation of monocytes into DCs expressing B7 and class II is facilitated by IFN-α in SLE (29). The pDC-derived type I IFNs directly affect the differentiation of Th1 and T follicular helper (Tfh) cells, both of which have been implicated in the pathogenesis of SLE (30, 31). Ag presentation by type I IFN-primed mature DCs causes CD4^+^T cell proliferation, leading to naïve B cell differentiation and autoAb production (28, 32). IFN-α also increases production of B cell-activating factor (BAFF) by pDCs and cDCs (16, 33). BAFF prolongs the survival of B cells and consequently promotes autoAb production via T cell-dependent and-independent mechanisms (34, 35). Collectively, pDC activation, followed by efficient Ag presentation by cDCs, underlies the immunopathogenesis of SLE. This consequence of DC activation seen in SLE is in contrast to that of type 1 AIP, in that cDC activation precedes that of pDCs in the latter autoimmunity.

## 5 Conclusions

The activation of pDCs producing type I IFNs underlies SLE and type 1 AIP immunopathogenesis. However, the phases of pDC involvement seem to be different in SLE and AIP: pDCs producing type I IFNs function as initiators rather than effectors in SLE, whereas the pathogenicity of pDCs is demonstrated in the effector phase rather than the induction phase in AIP. The clinical efficacy of biologics targeting pDCs has been verified in SLE patients (7, 8). The positive cytokine/chemokine feedback loop produced by cDCs, CD4^+^CXCR3^+^Th1 cells, and pDCs plays an essential role in developing murine and human AIP. Pharmacological intervention targeting chemokines produced by cDCs and T cells, such as CXCL9, CXCL10, and CCL25, may be beneficial for patients with type 1 AIP, in addition to pre-existing SLE biologics targeting pDCs and type I IFNs.
